# Safety profile of glucokinase activator AZD1656: systematic review and meta-analysis of 23 randomised trials in diabetic and healthy volunteers with relevance to immunomodulatory therapy

**DOI:** 10.1007/s10787-025-01785-z

**Published:** 2025-07-08

**Authors:** Mohamed M. Elsayed, Mohamed M. Abdalla

**Affiliations:** 1 Department of Clinical Pharmacy and Pharmacy Practice, Mansoura University, Mansoura, Egypt; 2 Department of Clinical Pharmacology, Glasgow University, Glasgow, UK

**Keywords:** Glucokinase enzyme, Glucokinase activator, AZD1656, Safety, Hypoglycaemia, Anti-diabetic, Immunomodulatory

## Abstract

Glucokinase enzyme plays a pivotal role in various physiological processes such as glucose metabolism, inflammation, and immunity. AZD1656 is a glucokinase activator (GKA) that shows proven efficacy in reducing blood glucose. It also possesses a marked interest in autoimmune diseases due to its immunomodulatory effect. Six databases were searched: PubMed, Scopus, the Cochrane Library, World of Science, ClinicalTrials.gov, and AstrazenecaClinicalTrials.com. This meta-analysis was conducted by following the Preferred Reporting Items for Systematic Reviews and Meta-Analyses (PRISMA). Twenty-three randomized controlled trials were included in this systematic review, and 19 studies were included in the meta-analysis. There was no significant difference between AZD1656 and placebo. Regarding total non-serious adverse events (a/e), the cumulative relative risk (RR) was 1.09 (95% CI 0.96–1.24, I^2^ = 30%, *p* = 0.19). The RR for low doses (< 100 mg), medium doses (≥ 100 and < 200 mg), and high doses (≥ 200 mg) were 1.17 (95% CI 0.98–1.40, I^2^ = 21%, *p* = 0.08), 1.19 (95% CI 0.96–1.48, I^2^ = 49%, *p* = 0.18), and 1.06 (95% CI 0.78–1.43, I^2^ = 34%, *p* = 0.72), respectively. For hypoglycaemic events, the cumulative RR was 2.03 (95% CI 0.94–4.39, I^2^ = 0%, *p* = 0.07). The RR for low doses, medium doses, and high doses were 2.59 (95% CI 0.59–11.43, I^2^ = 0%, *p* = 0.21), 2.48 (95% CI 0.80–7.72, I^2^ = 0%, *p* = 0.16), and 2.17 (95% CI 0.28–16.47, I^2^ = 0%, *p* = 0.46), respectively. The cumulative RR for serious a/e was 0.85 (95% CI 0.21–3.48, I^2^ = 0%). AZD1656 is a well-tolerated, safe glucokinase activator. It has promising potential as an anti-diabetic and immunomodulatory agent, supporting its further investigation in immunomodulatory and inflammatory diseases.

## Introduction

The glucokinase enzyme is a subtype of hexokinases expressed mainly in liver and pancreatic β-cells ​(Matschinsky 2009)​. Glucokinase activation shows a hypoglycaemic effect by catalyzing the rate-limiting step in glucose metabolism, converting glucose to glucose-6-phosphate ​(Iynedjian 2009). It acts as a glucose sensor and regulates glucose-dependent insulin secretion and glucose uptake by hepatic cells ​(Matschinsky 2009)​. Moreover, although recent studies showed that glucokinase is also expressed in gut endocrine cells at low levels, it has no role in glucose homeostasis ​(Murphy et al. 2009)​. Furthermore, the glucokinase enzyme plays a pivotal role in immunity via regulating glycolysis, which enhances the migration of regulatory T cells to inflamed tissues ​(Kishore et al. 2018)​. This indicates a potential role of glucokinase activators in immunopathological diseases and in lowering blood glucose levels.

AZD1656 is a novel glucokinase activator (GKA) investigated for safety in multiple trials in healthy or diseased volunteers. It has proven efficacy in lowering blood glucose levels ​(Kiyosue et al. 2013)​. The immuno-regulatory effect of AZD1656 was investigated in a COVID-19 phase II trial ​(Chorlton et al. 2022)​. Upcoming studies will examine the impact of AZD1656 on auto-immune diseases, particularly lupus. AZD1656 is an appealing target for future research, yet no previous meta-analysis studied its safety in healthy and diseased populations.

## Materials and methods

### Search strategy

Literature search, study design, and data analysis were performed following Preferred Reporting Items for Systematic Reviews and Meta-Analyses (PRISMA) guidelines ​(Moher et al. 2009)​. The literature search was conducted by two independent reviewers in PubMed, Scopus, the Cochrane Library, World of Science, ClinicalTrials.gov, and AstrazenecaClinicalTrials.com with the following keywords or terms: "AZD1656" OR "glucokinase activator" AND "Diabetes mellitus" OR “type II diabetes” OR "T2DM" OR "Hypoglycaemia" OR "Safety" OR "Healthy".

### Inclusion and exclusion criteria

The Main inclusion criteria were as follows: (1) patients: We included trials of any gender with a diagnosis of T2DM or Healthy (18 years of age or older); (2) interventions: any dose of AZD1656 used as monotherapy or combination therapy; (3) comparison: placebo or active comparators with or without background therapy; (4) outcomes: at least one of the following indicators was reported Total non-serious a/e, serious a/e, and hypoglycaemia; (5) study design: randomized controlled trials (RCTs).

The studies were excluded based on the following exclusion criteria: (1) contains patients of age under 18 or over 75 years; (2) non-randomized control trials; (3) contains less than 10 population.

### Quality assessment

The quality of the included studies was evaluated using the Cochrane risk-of-bias tool for randomized trials, which assesses (1) the randomization process,(2) allocation concealment, (3) blinding of participants and personnel, (4) blinding of outcome assessors assessments, (5) incomplete outcome data, (6) selective outcome reporting, (7) Other potential sources of bias and give overall bias assessment as ‘low’, ‘high’, or ‘some concern’ according to Algorithm for suggested judgment of risk of bias.

### Data extraction

Two independent researchers collected the following data. Both reviewed each eligible study, and the following information was extracted: (1) study characteristics (name of first author, year of publication, country, number of population); (2) patient characteristics (gender, mean or range of age, disease status); (3) intervention (dosage, duration of intervention, comparator); (4) outcome measures.

### Statistical analysis

The safety of GKA AZD1656, compared to placebo, was assessed based on three major criteria: serious adverse events (a/e), total non-serious a/e, and hypoglycaemia. Reporting hypoglycaemic events was based on measuring plasma glucose levels, less than 70 mg/dL or 3.9 mmol/L. Additional analyses were performed to assess the safety profile of AZD1656 with respect to total non-serious a/e and hypoglycaemic episodes, stratified by total daily dose analysis. Studies were classified based on the dosing threshold: a total daily dose < 100 mg was categorized as low dose, doses ranging from ≥ 100 and < 200 mg were identified as medium dose, and doses ≥ 200 mg were classified as high dose. If a trial had different subgroups of doses and results were provided for each subgroup dose, each dose was included as a separate study in the meta-analysis (Kiyosue et al. 2013; Morrow et al. 2012; Wilding et al. 2013). However, if data were not provided for each dose of the study, the study was categorized based on the highest or targeted dose after titration according to the previous criteria. Additionally, cumulative meta-analyses were conducted by statistically pooling all studies irrespective of the dose; results from different sub-doses within the same trial were aggregated into a single dataset and compared against placebo. This approach was adopted to alleviate potential overestimation of the placebo effect size that would have resulted from combining effect sizes of different sub-dose analyses.

Analysis was performed via the Meta package of software R version 4.4.1 ​(Balduzzi et al. 2019)​. Relative Risk (RR) between AZD1656 events and placebo events was utilized as a safety outcome indicator. Heterogeneity between studies was evaluated based on the I^2^ parameter. The fixed-effect model was used to synthesize the RR outcome for heterogeneity ≤ 50%; otherwise, the random-effect model was used. A sensitivity analysis was conducted to test the consistency of the results. First, the analysis was re-performed excluding studies with high heterogeneity. Then, a stepwise exclusion of studies was performed until there were only two studies. Furthermore, for another sensitivity analysis, the whole analysis was repeated using odds ratio (OR) instead of RR and random effect method instead of fixed effect. A subgroup analysis was conducted to test AZD1656 safety among different study characteristics. Safety was investigated in trials in healthy volunteers, DM II volunteers, and among trials with continuous oral doses of AZD1656. A 95% confidence interval was used to report each outcome.

## Results

### Study selection and characteristics

**Fig. 1:**
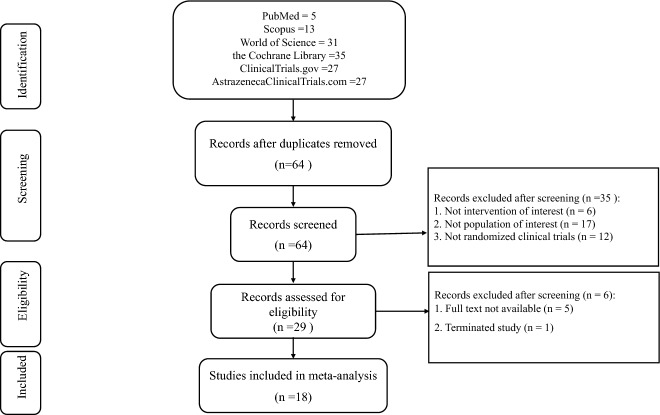
Literature search results in accordance with PRISMA reporting

A total of 138 studies were retrieved through a database search from six databases and 64 duplicates were excluded. After reviewing the titles and abstracts for inclusion and exclusion criteria, 34 articles were excluded and then 28 articles were left for full-text evaluation. After a complete assessment of the 28 articles, one article was excluded because it had been terminated and had no results, and five articles were without full text. Finally, 23 studies met our inclusion criteria and were considered for systematic review and meta-analysis. Of these, 19 were included in the meta-analysis. Figure 1 shows the flow chart of research selection, and the characteristics of the included studies are represented in Table 1.

**Table 1:** Characteristics of included studies

Study	Country	Phase	Population	M/F	Age (mean, range)	Intervention	Duration (weeks)	Comparison group	Disease status	Reported outcomes
Chorlton et al. (2022)	Multiple countries	II	153	97/56	64.3 (≥ 18)	100 mg orally twice daily	3	Placebo	COVID-19 with either DM I or II	Mortality, length of hospitalization, serious a/e, and total a/e
Kiyosue et al. (2013)	Japan	II	224	192/32	56	Low doses (10–80 mg), middle doses (20–140 mg), high doses (40–200 mg)	16	Placebo	DM II	Total non-serious a/e, serious a/e, and hypoglycaemia
Morrow et al. (2012)	USA	I	52	41/11	52.3 (37–75)	7, 20, 40, or 80 mg orally twice daily	4	Placebo	DM II	Total non-serious a/e, serious a/e, and hypoglycaemia
Norjavaara et al. (2012)	Sweden	I	12	12/0	20–45	40 mg or 80 mg single oral dose	Single dose	Insulin	Healthy	Total non-serious a/e, serious a/e, and hypoglycaemia
Wilding et al. (2013)	Multiple countries	II	530	268/262	56 (20–81)	20 mg fixed-dose, 40 mg fixed-dose, 10–140 mg, and 20–200 mg oral total daily doses	24	Placebo	DM II	Total non-serious a/e, serious a/e, and hypoglycaemia
NCT00817778	USA	IIA	27	16/11	60.2 (34–74)	Multiple tolerable oral doses twice daily to reach a target daily dose of 100 mg	4	Placebo	DM II	Total non-serious a/e, serious a/e, and hypoglycaemia
NCT00856908	USA	IIA	20	8/12	53.8 (40–69)	Multiple tolerable oral doses twice daily to reach a target daily dose of 100 mg	4	Placebo	DM II	Total non-serious a/e, serious a/e, and hypoglycaemia
D1020C00001	USA	I	32	NA	42.5 (20–45)	Single oral dose from 2 mg to 180 mg	Single dose	Placebo	Healthy	Serious a/e
D1020C00003	USA	I	36	36/0	(20–40)	6 mg, 18 mg, 30 mg, 50 mg, 100 mg, or 180 mg orally once daily	4.6	Placebo	Healthy	Total non-serious a/e, serious a/e, and hypoglycaemia
D1020C00004	Japan	I	24	NA	45 (30–75)	20 mg, 40 mg, or 80 mg orally twice daily	1.1	Placebo	DM II	Total non-serious a/e, serious a/e, and hypoglycaemia
D1020C00010	USA	I	12	10/2	(20–65)	90 mg single oral dose	Single dose	Placebo	DM I	Serious a/e and hypoglycaemia
D1020C00014	USA	I	30	NA	(30–75)	10 mg, 30 mg, 90 mg, or 150 mg orally twice daily	1	Placebo	DM II	Total non-serious a/e, serious a/e, and hypoglycaemia
D1020C00015	USA	I	26	19/7	52.5(30–75)	5 mg, 12.5 mg, 25 mg, or 50 mg orally twice daily	1.4	Placebo	DM II	Total non-serious a/e, serious a/e, and hypoglycaemia
D1020C00017	USA	I	36	NA	52.3 (37–75)	80 mg orally once daily or 40 mg orally twice daily	0.57	Dosing regimens	DM II	Total non-serious a/e, serious a/e, and hypoglycaemia
D1020C00026	USA	I	21	16/5	45 (30–75)	12.5 mg, 40 mg, or 100 mg orally twice daily	1.3	Placebo	DM II	Total non-serious a/e and serious a/e
D1020C00027	UK	I	16	NA	≥ 18	20 mg or 50 mg orally twice daily	1.4	Placebo	DM II	Total non-serious a/e, serious a/e, and hypoglycaemia
D1020C00028	USA	I	26	15/11	≥ 18	20 mg or 40 mg orally twice daily	8	Placebo	DM II	Total non-serious a/e, serious a/e, and hypoglycaemia
D1020C00029	USA	I	44	31/15	58	20 mg orally twice daily	1.7	Dosing regimens	DM II	Total non-serious a/e, serious a/e, and hypoglycaemia
D1020C00030	Sweden	I	12	7/5	46.5 (18–75)	20 mg orally once daily	8	Placebo	DM II	Total non-serious a/e, serious a/e, and hypoglycaemia
D1020C00031	USA	I	20	NA	≥ 18	40 mg or 100 mg orally once daily	1	Placebo	DM II	Total non-serious a/e, serious a/e, and hypoglycaemia
D1020C00032	Germany	I	12	NA	46.5 (18–75)	40 mg or 100 mg orally twice daily	8	Sitagliptin	DM II	Total non-serious a/e, serious a/e, and hypoglycaemia
D1020C00033	USA	I	20	NA	≥ 18	50 mg orally twice daily before food or 100 mg orally once daily before and after food	8	Dosing Regimens	DM II	Total non-serious a/e, serious a/e, and hypoglycaemia
D1020C00044	USA	I	24	24/0	50 (20–60)	150 mg, 300 mg, or 450 mg single oral doses	Single dose	Placebo	DM II	Total non-serious a/e, serious a/e, and hypoglycaemia

### Quality assessment

**Fig. 2:**
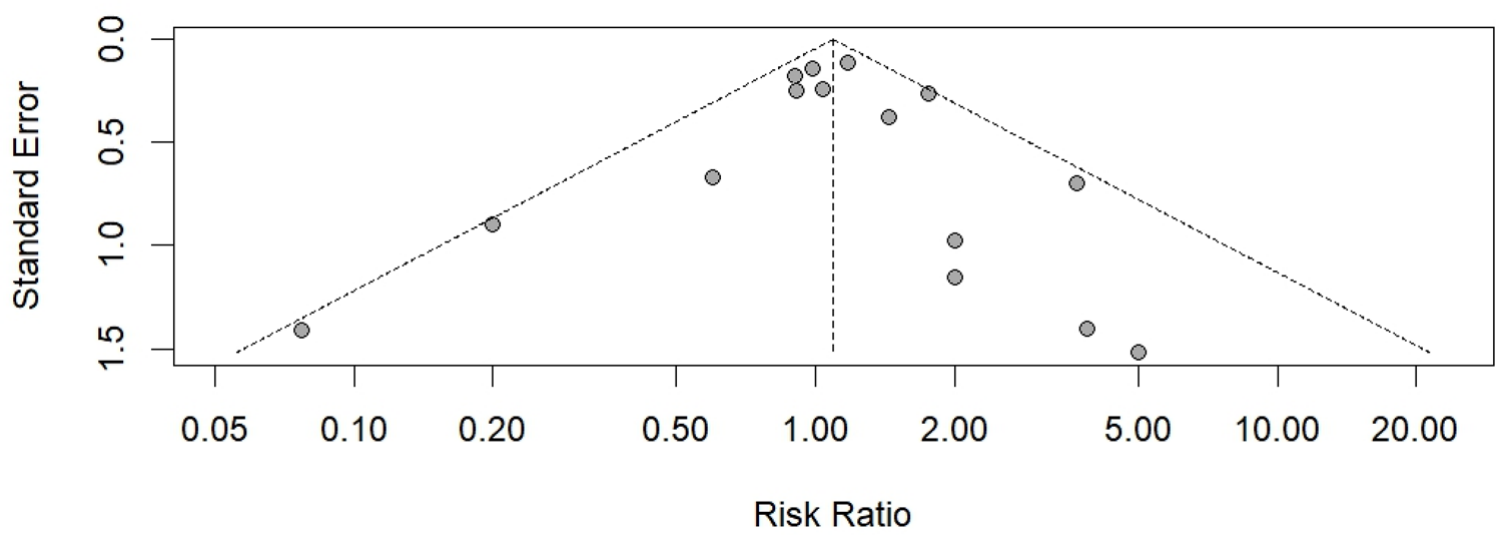
Funnel plot assessment of publication bias

The quality of the eligible studies was assessed using the Cochrane risk-of-bias tool for randomized trials (ROB 2.0), A summary table of ROB 2.0 is available in the supplementary file. All 23 trials had randomly allocated participants. Random sequence generation, attrition bias, and reporting bias were judged to be at low risk in 18 studies (78%) and at some concern in 5 studies (22%). None of the trials showed differences in baseline characteristics between the combination therapy and AZD1656 groups. Deviations from the intended interventions were judged to be at low risk in 20 studies (87%), at some concern in two studies (9%), and high risk in one study (4%). Incomplete outcome data were judged to be at low risk in 15 studies (65%), at some concern in 2 studies (9%), and high risk in 6 studies (26%). Measurement of the outcome was judged to be at low risk in 14 studies (61%), at some concern in 2 studies (9%), and high risk in 7 studies (30%). Selective outcome reporting was judged to be at low risk in 19 studies (82%), at some concern in 2 studies (9%), and high risk in 2 studies (9%). Regarding publication bias, we deduced low risk after visual evaluation of the funnel plot, as shown in Fig. 2. Additionally, the p-value of Egger’s test was 0.84, indicating no significant asymmetry. The risk of bias assessment for each included study is shown in the Supplementary File.

### Studies classification according to dose

Three studies (Kiyosue et al. 2013; Morrow et al. 2012; Wilding et al. 2013) reported safety outcomes for multiple doses of AZD1656. Consequently, their results were categorized into separate analyses based on dose, following predefined criteria. Additionally, four studies (Malmberg 2009b; Norjavarra et al. 2012; Skrtic 2010b, e) were classified as low-dose, eight as medium-dose (DeNoia et al. 2012; Hompesch et al. 2012; Malmberg 2008a, b, 2009d, 2010b; Skrtic 2010a, d), and four as high dose (Malmberg 2009c, 2010a; Morrow 2011; Skrtic 2010c).

### Total non-serious a/e

**Fig. 3:**
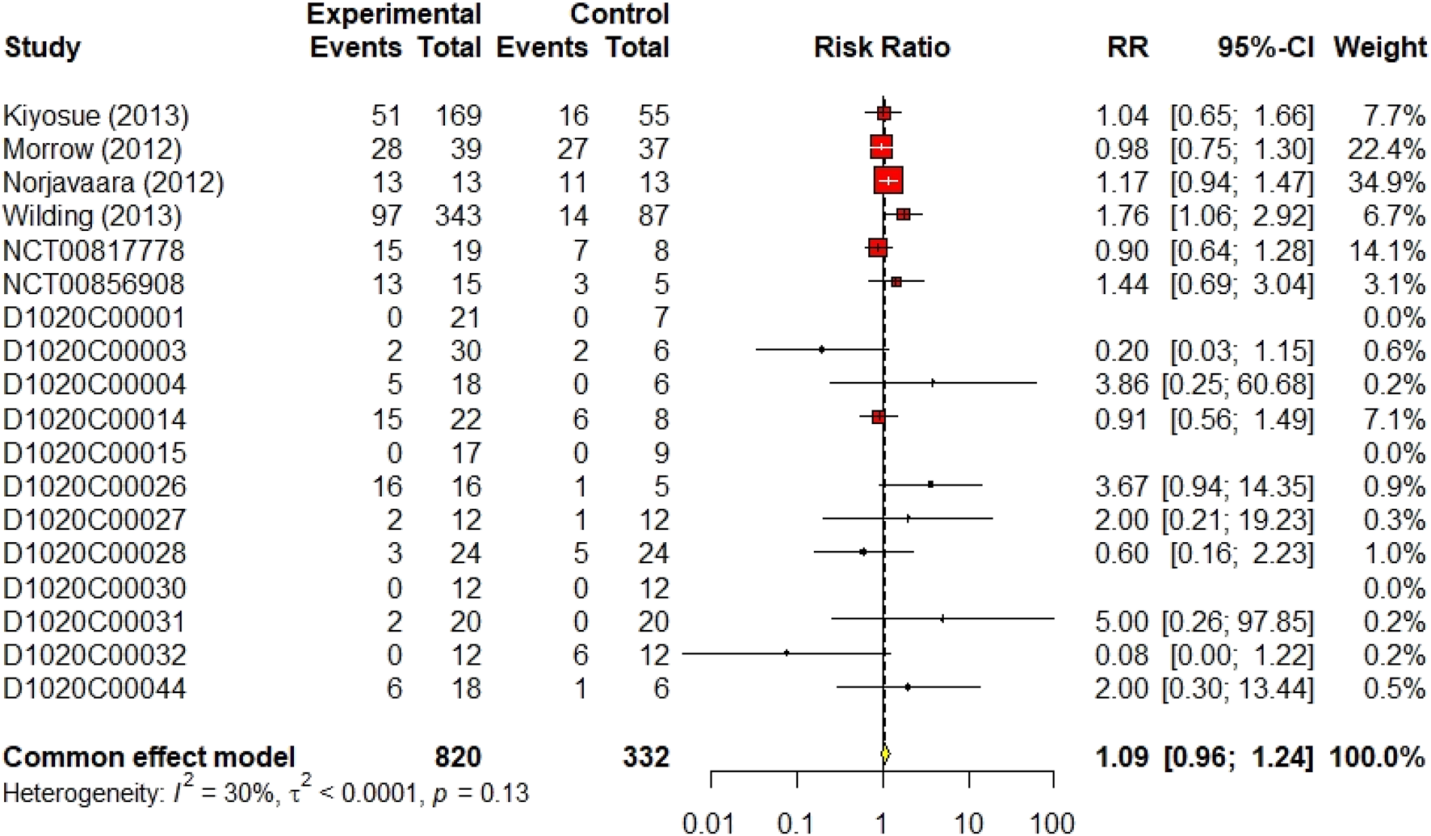
Forest plot of cumulative total non-serious events

In 18 studies (DeNoia et al. 2012; Hompesch et al. 2012; Kiyosue et al. 2013; Malmberg 2008b, 2009b, c, 2009d, 2010a, b; Morrow 2011; Morrow et al. 2012; Norjavaara et al. 2012; Skrtic 2010a, b, c, d, e; Wilding et al. 2013), a total of 820 patients received AZD16565, resulting in 263 events (32%). Meanwhile, 332 patients in the control group experienced 100 events (30%), and since heterogeneity was 30%, we used the fixed-effect model to synthesize the RR outcome, The RR was 1.09 (95% CI 0.96–1.24, I^2^ = 30%, *p* = 0.19). The forest plot for cumulative total non-serious a/e is shown in Fig. 3.

**Fig. 4:**
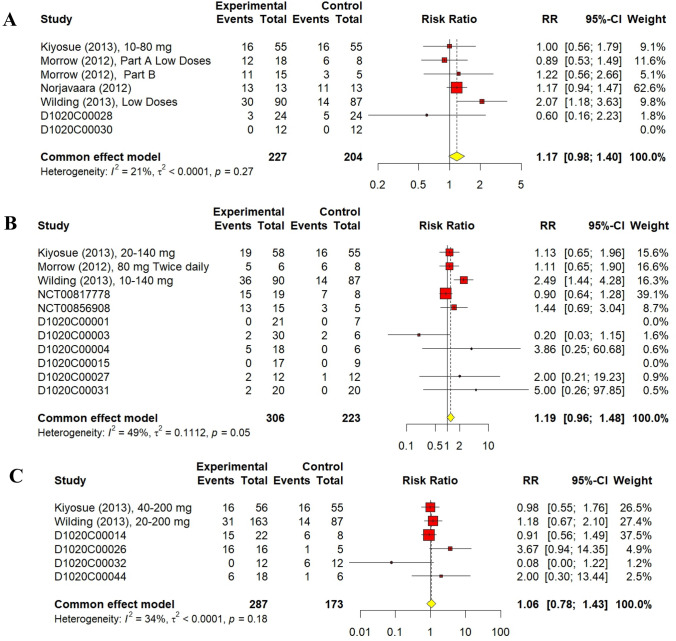
Forest plots of total non-serious events classified by dose. (**A**) Forest plot of total non-serious events of low doses AZD1656 (< 100 mg daily). (**B**) Forest plot of total non-serious events of medium doses AZD1656 (≥ 100 and < 200 mg daily). (**C**) Forest plot of total non-serious events of high doses AZD1656 (≥ 200 mg daily)

A total of 227 volunteers received low doses of AZD1656 with 85 events encountered (37.44%). Simultaneously, 204 volunteers received placebo with 55 events encountered (26.96%). The RR for volunteers who received low doses of AZD1656 was 1.17 (95% CI 0.98–1.40, I^2^ = 21%, *p* = 0.08). Moreover, 306 volunteers received medium doses of AZD1656 with 99 events experienced (32.35%), while 223 volunteers received placebo with 49 events experienced (21.97%). The RR for volunteers who received medium doses of AZD1656 was 1.19 (95% CI 0.96–1.48, I^2^ = 49%, *p* = 0.18). Regarding high doses, 287 participants were administered various AZD1656 doses ≥ 200 mg with 84 events reported (29.27%), compared to 173 participants in the placebo group where 44 events were reported (25.43%). The RR associated with AZD1656 high doses was 1.06 (95% CI 0.78–1.43, I^2^ = 34%, *p* = 0.72). The sub-dose analyses of total non-serious events are shown in Fig. 4. Both cumulative analysis of total non-serious events and sub-dose analyses indicated no significant increase compared to placebo.

### Serious a/e

**Fig. 5:**
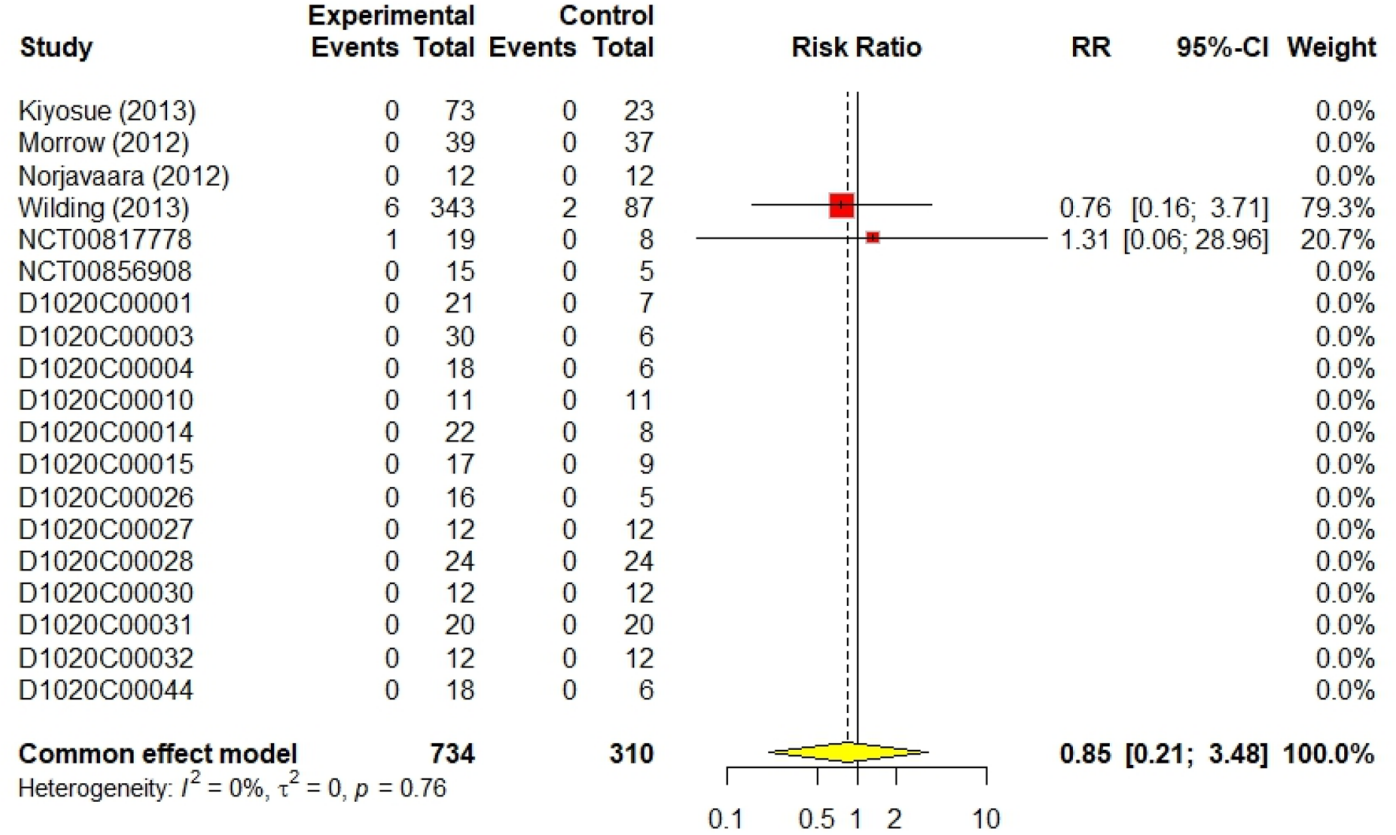
Forest plot of cumulative serious events

Of the 734 patients who received AZD1656 in the 19 included studies (DeNoia et al. 2012; Hompesch et al. 2012; Kiyosue et al. 2013; Malmberg 2008a, b; 2009b, c, d; 2010a, b; Morrow 2011, Morrow et al. 2012; Norjavaara et al. 2012; Skrtic 2010a, b, c, d, e; Wilding et al. 2013), only 7 developed serious adverse events (0.7%). The control group of 310 patients experienced a similarly low rate of serious adverse events, with only 2 cases reported (0.6%), The RR for serious adverse events was 0.85 (95% CI 0.21–3.48, I^2^ = 0%), indicating no significant difference between the AZD1656 group and the control group. The forest plot for cumulative serious a/e is shown in Fig. 5.

### Hypoglycaemia

**Fig. 6:**
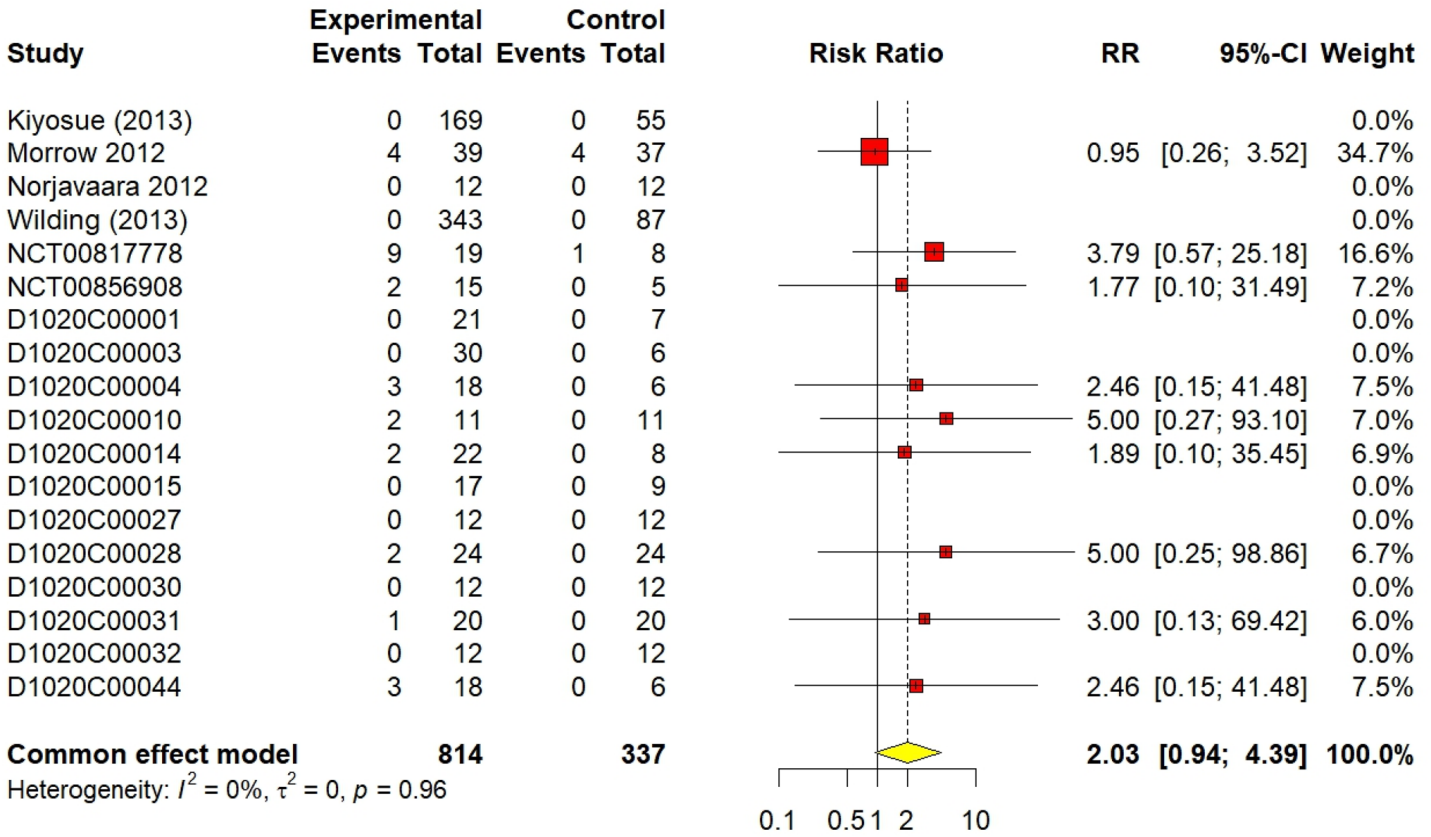
Forest plot of cumulative hypoglycaemic events

A total of 18 studies were included (DeNoia et al. 2012; Hompesch et al. 2012; Kiyosue et al. 2013; Malmberg 2008a, b; 2009b, c, d; 2010b; Morrow 2011; Morrow et al. 2012; Norjavaara et al. 2012; Skrtic 2010a, b, c, d, e; Wilding et al. 2013), with 9 studies reporting zero hypoglycaemic events for AZD1656 and placebo. The AZD1656 group (n = 814) experienced 28 hypoglycaemic events (3.4%), compared to 5(1.4%) in the control group (n = 337), The RR for hypoglycaemia was 2.03 (95% CI 0.94–4.39, I^2^ = 0%, *p* = 0.07), suggesting a non-significant increased risk of hypoglycaemia in the AZD1656 group compared to the control group. The forest plot for cumulative hypoglycaemic events is shown in Fig. 6.

**Fig. 7:**
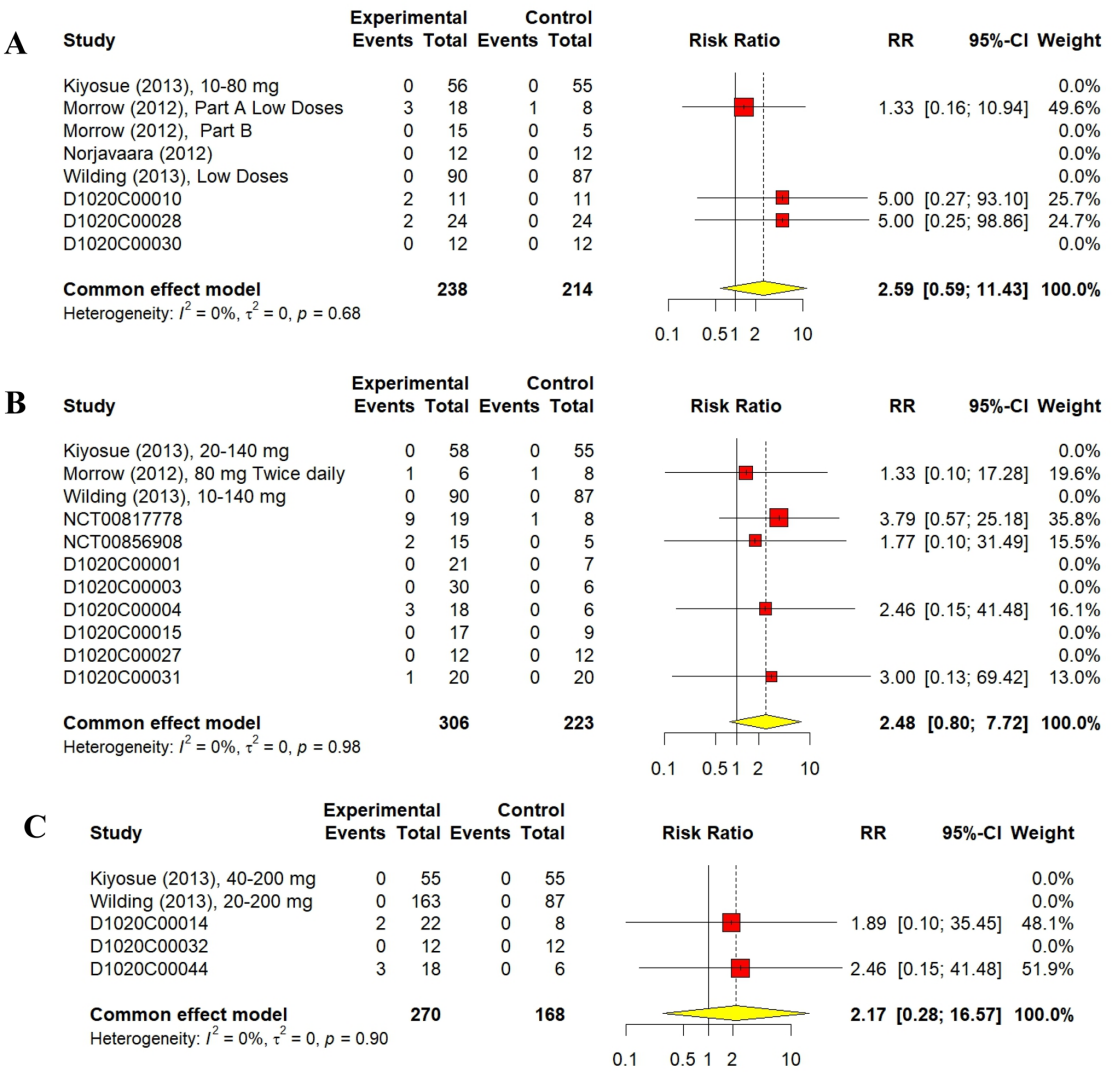
Forest plot of hypoglycaemic events classified by dose. (**A**) Forest plot of hypoglycaemic events of low doses AZD1656 (< 100 mg daily). (**B**) Forest plot of hypoglycaemic events of medium doses AZD1656 (≥ 100 and < 200 mg daily). (**C**) Forest plot of hypoglycaemic events of high doses AZD1656 (≥ 200 mg daily)

A total of 238 volunteers received low doses of AZD1656 with only seven hypoglycaemic episodes encountered (2.94%). Simultaneously, 214 volunteers received placebo with one episode encountered (0.47%). The RR of hypoglycaemic episodes for volunteers who received low doses of AZD1656 was 2.59 (95% CI 0.59–11.43, I^2^ = 0%, *p* = 0.21). Furthermore, Medium doses of AZD1656 were administered to 306 volunteers with 16 events experienced (5.23%), while 223 volunteers were administered a placebo with two events experienced (0.09%). The hypoglycaemic RR for volunteers who received medium doses of AZD1656 was 2.48 (95% CI 0.80–7.72, I^2^ = 0%, *p* = 0.16). High doses were delivered to 270 participants with five events reported (1.85%), compared to 168 participants in the placebo group where no events were reported (0%). The RR associated with AZD1656 high doses was 2.17 (95% CI 0.28–16.47, I^2^ = 0%, *p* = 0.46). The sub-dose analyses of hypoglycaemic events are shown in Fig. 7. Both cumulative analysis and sub-dose analyses indicated no significant increase in hypoglycaemic events compared to placebo.

## Discussion

This meta-analysis investigated the safety of GKA AZD1656 in terms of total non-serious a/e, serious a/e, and hypoglycaemia due to various potential upcoming applications of GKA in diabetes and auto-immune diseases. We aimed to provide reliable data about AZD1656 safety for future trials. Based on doses used in the included studies, our meta-analysis revealed that GKA AZD1656 was safe from 6 (Malmberg 2008b) to 300 mg (Malmberg 2009c) total daily dose, and from 2 (Malmberg 2008a) to 450 mg (Morrow 2011) single oral dose.

The results of 18 randomized controlled trials (RCTs) were analysed using the fixed-effect method for total non-serious a/e outcomes. There was no significant difference between AZD1656 and placebo; the cumulative RR was 1.09 (95% CI 0.96–1.24, I^2^ = 30%, *p* = 0.19). Similarly, non-serious adverse events of low-dose (< 100 mg), medium-dose (≥ 100 and < 200 mg), and high-dose (> 200 mg) groups did not significantly increase from the placebo group; their RRs were 1.17 (95% CI 0.98–1.40, I^2^ = 21%, *p* = 0.08), 1.19 (95% CI 0.96–1.48, I^2^ = 49%, *p* = 0.18), and 1.06 (95% CI 0.78–1.43, I^2^ = 34%, *p* = 0.72), respectively. For hypoglycaemic events, AZD1656 was entirely safe compared to placebo. Nine studies from 18, comprising 840 volunteers, reported no hypoglycaemic events encountered with either AZD1656 or placebo. The cumulative RR for hypoglycaemia was 2.03 (95% CI 0.94–4.39, I^2^ = 0%, *p* = 0.07), indicating no significant difference. The RR for low doses, medium doses, and high doses were 2.59 (95% CI 0.59–11.43, I^2^ = 0%, *p* = 0.21), 2.48 (95% CI 0.80–7.72, I^2^ = 0%, *p* = 0.16), and 2.17 (95% CI 0.28–16.47, I^2^ = 0%, *p* = 0.46), respectively. Regarding serious a/e, only two trials had patients who experienced serious a/e. A total of nine patients, 7 patients treated with AZD1656 and 2 patients treated with placebo, experienced serious a/e. The RR was 0.85 (95% CI 0.21–3.48, I^2^ = 0%). Reported serious a/e in volunteers treated with AZD1656 were as follows: rapid atrial fibrillation (DeNoia et al. 2012), cerebrovascular accident (Wilding et al. 2013, 20–200 mg dose), increased blood creatine phosphokinase (Wilding et al. 2013, 20–200 mg dose), diabetic foot (Wilding et al. 2013, 10–140 mg), haemorrhagic intestinal Diverticulum (Wilding et al. 2013, 40 mg fixed dose), unstable angina (Wilding et al. 2013, 20 mg fixed dose), and anal abscess (Wilding et al. 2013, open-label).

Regarding total non-serious a/e, five trials (DeNoia et al. 2012; Hompesch et al. 2012; Kiyosue et al. 2013; Morrow et al. 2012; Wilding et al. 2013) reported the detailed adverse events encountered in diabetic patients administered AZD1656. The most observed event was nasopharyngitis; 43 patients comprising 9% of the population suffered from this infection. The second most encountered events were diarrhea and headache with a percentage of 5.4% and 5.2%, respectively. The complete list of adverse events is listed in Table 2. Two studies (Malmberg 2008b; Norjavaara et al. 2012) reported complete adverse events in healthy volunteers; hunger was the most reported adverse event.

**Table 2:** Total non-serious adverse events encountered with diabetic volunteers treated with AZD1656

Event	Number of patients suffered (percent)
*Cardiac disorders*	
Supraventricular tachycardia	1 (0.21%)
*Eye disorders*	
Asthenopia	2 (0.42%)
*Gastrointestinal disorders*	
Constipation	5 (1.05%)
Diarrhea	26 (5.44%)
Gastritis	5 (1.05%)
Nausea	12 (2.51%)
Toothache	1 (0.21%)
Upper abdominal pain	1 (0.21%)
Vomiting	7 (1.46%)
*General disorders*	
Asthenia	7 (1.46%)
Non-cardiac chest pain	1 (0.21%)
*Infections and infestations*	
Bronchitis	3 (0.63%)
Gastroenteritis	4 (0.84%)
Nasopharyngitis	43 (9.0%)
Pharyngitis	3 (0.63%)
Upper respiratory tract infection	5 (1.05%)
Urinary tract infection	2 (0.42%)
*Injury, poisoning, and procedural complications*	
Application site reaction	2 (0.42%)
Catheter site pain	2 (0.42%)
Skin laceration	1 (0.21%)
*Investigations*	
Alanine aminotransferase increased	3 (0.63%)
Aspartate aminotransferase increased	4 (0.84%)
Blood triglycerides increased	2 (0.42%)
Electrocardiogram T wave inversion	2 (0.42%)
*Metabolism and nutrition disorders*	
Blood glucose decreased	15 (3.14%)
Hyperglycaemia	5 (1.05%)
Peripheral oedema	1 (0.21%)
*Musculoskeletal and connective tissue disorders*	
Back pain	4 (0.84%)
Limb discomfort	1 (0.21%)
Pain in the extremities	9 (1.88%)
*Nervous system disorders*	
Dizziness	16 (3.35%)
Headache	25 (5.23%)
Restless leg syndrome	1 (0.21%)
Tremors	12 (2.51%)
*Respiratory, thoracic, and mediastinal disorders*	
Allergic rhinitis	1 (0.21%)
Oropharyngeal pain	1 (0.21%)
Rhinorrhoea	2 (0.42%)
Upper respiratory tract inflammation	5 (1.05%)
*Skin and subcutaneous tissue disorders*	
Asteatosis eczema	1 (0.21%)
Erythema	1 (0.21%)
Hyperhidrosis	9 (1.88%)
Rash	1 (0.21%)
Skin irritation	1 (0.21%)
*Vascular disorders*	
Ecchymosis	1 (0.21%)

We performed a sensitivity analysis to test the consistency of our results regarding the total non-serious a/e. The whole analysis was re-performed using different parameters, OR instead of RR and random effect model instead of fixed effect model. The OR did not differ significantly from the RR. Using the fixed effect model, The OR for total non-serious a/e was 1.32 (95% CI 0.93–1.89, I^2^ = 42%, *p* = 0.12), while it was 1.28 (95% 0.81–2.01, I^2^ = 42%, *p* = 0.29). The RR of total non-serious a/e using the random effect model was 1.09 (95% CI 0.96–1.24, I^2^ = 30%, *p* = 0.19). Furthermore, another sensitivity analysis was performed. First, we excluded highly heterogeneous studies. Then, one more study was excluded at each step of the analysis. The results of the analysis affirmed the consistency of the primary results. The sensitivity analysis is provided in the supplementary file.

**Fig. 8:**
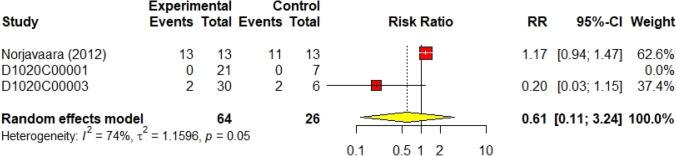
Forest plot of the safety of AZD1656 in healthy volunteers

**Fig. 9:**
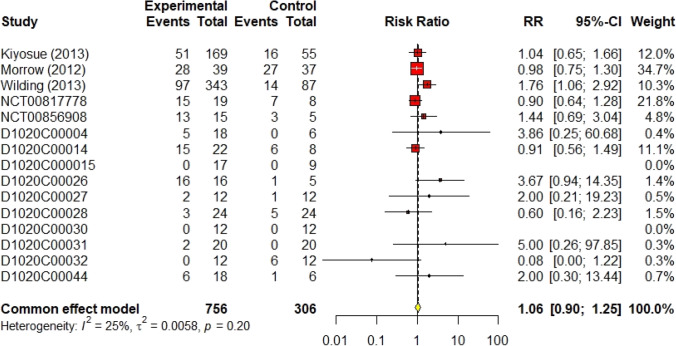
Forest plot of the safety of AZD1656 in diabetic volunteers

**Fig. 10:**
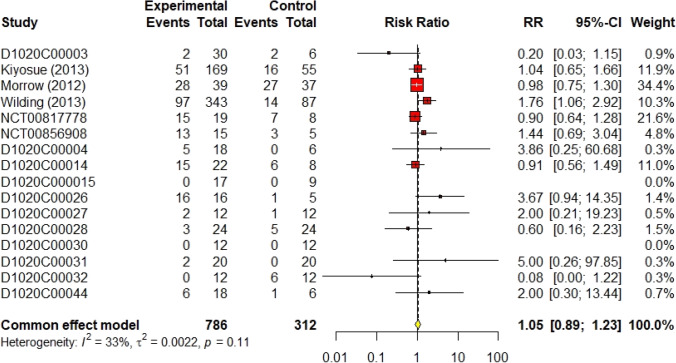
Forest plot of the safety of oral multiple doses of AZD1656

The results of the sub-group analysis did not differ significantly from the main analysis. Three trials (Malmberg 2008a, b; Norjavaara et al. 2012) investigated the safety of AZD1656 in healthy volunteers with a population of 90 volunteers. As shown in Fig. 8, the RR of total non-serious a/e in healthy volunteers was 0.61 (95% CI 0.11–3.24, I^2^ = 74%) using the random effect model. Moreover, AZD1656 was completely safe in patients with DM II. Fifteen trials (DeNoia et al. 2012; Hompesch et al. 2012; Kiyosue et al. 2013; Malmberg 2008b; 2009b, c; 2010b; Morrow 2011; Morrow et al. 2012; Wilding et al. 2013; Skrtic 2010a, b, c, d, e) studied the safety of AZD1656 in diabetic patients with a population of 1026 volunteers; the RR was 1.06 (95% CI 0.90–1.25, I^2^ = 25%), shown in Fig. 9. Sixteen studies (DeNoia et al. 2012; Hompesch et al. 2012; Kiyosue et al. 2013; Malmberg 2008b; 2009d; D1020C00010; Malmberg 2009c; 2010b; Morrow 2011; Morrow et al. 2012; Wilding et al. 2013; Skrtic 2010a, b, c, d, e) reported the efficacy of AZD1656 as continuous oral doses comprising 1098 volunteers. The RR was 1.05 (95% CI 0.89–1.23, I^2^ = 33%), shown in Fig. 10. All sub-group analyses concluded that AZD1656 is a safe, oral GKA.

Yang and his colleagues reported that AZD1656 has a significantly higher risk of hypoglycaemia (OR = 18.100, 95% CI 2.438 to 134.406, *P* = 0.005; I^2^ = 0.0%) (Yang 2023). This different conclusion was due to the inconsistency in Yang’s meta-analysis since it was only based on two trials, which were also included in our study. Upon increasing the sample size and number of included trials, 18 RCTs, we concluded that AZD1656 is completely safe compared to placebo.

We addressed some limitations while working on this meta-analysis. First, some of the included trials were single-blinded and open-labelled which had a higher risk of reporting bias; however, this issue was resolved by conducting a sensitivity study. Second, due to the available data reported from each study, sub-group analysis based on individual doses was unattainable. Third, we could not include studies that investigated the immunomodulatory effect of AZD1656 in this meta-analysis since they were non-randomized; nevertheless, the current study showed that AZD1656 was safe and well-tolerated. Further randomized controlled trials should be conducted to further investigate the effect of AZD1656 in auto-immune and inflammatory diseases.

## Conclusion

This extensive meta-analysis proved that AZD1656 is a well-tolerated and safe glucokinase activator based on the results of 19 randomized controlled trials. AZD1656 represents a potential therapeutic alternative in diabetes, autoimmune, and inflammatory diseases. Further trials are required to test AZD1656 in these diseases.

## Data Availability

Not applicable.

## Abbreviations

GKA: Glucokinase activator
OR: Odds ratio
RR: Relative risk
